# Strong recommendations from low certainty evidence: a cross-sectional analysis of a suite of national guidelines

**DOI:** 10.1186/s12874-023-01895-8

**Published:** 2023-03-25

**Authors:** Ming Chuen Chong, Melissa K. Sharp, Susan M. Smith, Michelle O’Neill, Máirín Ryan, Rosarie Lynch, Kamal R. Mahtani, Barbara Clyne

**Affiliations:** 1 Department of General Practice, RCSI University of Medicine and Health Sciences, Dublin, Dublin 2 Ireland; 2 Department of Public Health and Primary Care, School of Medicine, Trinity College Dublin, Dublin, Dublin 2 Ireland; 3 Health Information and Quality Authority, George’s Court, George’s Lane, Dublin, Dublin 7 Ireland; 4 Department of Health, Clinical Effectiveness and Antimicrobial Resistance Unit, National Patient Safety Office, Dublin, Ireland; 5 Nuffield Department of Primary Care Health Sciences, University of Oxford, Oxford, England

**Keywords:** Clinical guidelines, Evidence-based medicine, Certainty of the evidence, Strength of recommendations, GRADE

## Abstract

**Background:**

Clinical guidelines should be based on a thorough evaluation of the evidence and generally include a rating of the quality of evidence and assign a strength to recommendations. Grading of Recommendations Assessment, Development and Evaluation (GRADE) guidance warns against making strong recommendations when the certainty of the evidence is low or very low, but has identified five paradigmatic situations (e.g. life-threatening situations) where this may be justified.

**Aims and objectives:**

We aimed to characterize the strength of recommendations and certainty of the evidence in Irish National Clinical Guidelines using the GRADE approach.

**Methods:**

All National Clinical Guidelines from the National Clinical Effectiveness Committee (NCEC) website using the GRADE approach (fully or partially) were included. All recommendations and their corresponding certainty of the evidence, strength of recommendations and justifications were extracted. Authors classified instances of strong recommendations with low certainty evidence (referred to as discordant recommendations) into one of the five paradigmatic situations. Descriptive statistics were calculated.

**Results:**

From the 29 NCEC Clinical Guidelines available at the time of analysis, we identified 8 guidelines using GRADE with a total of 240 recommendations; 38 recommendations did not use the GRADE approach and were excluded. Half of the included guidelines focused on emergency situations. In the final dataset of 202 recommendations, 151 (74.7%) were classified as strong and 51 (25.3%) as conditional. Of the 151 strong recommendations, 55 (36.4%) were supported by high or moderate certainty evidence and 96 (63.6%) by low or very low certainty evidence and were considered discordant. Of these 96 discordant recommendations, 55 (73.7%) were consistent with one of the five paradigmatic situations. However, none were specifically described as such within the guidelines.

**Conclusions:**

The proportion of discordant recommendations identified in this analysis was higher than some previous international studies (range of all strong recommendations being discordant 30–50%), but similar to other guidelines focused on emergency situations. The majority of discordant recommendations could be mapped to one of the five situations, but no National Clinical Guideline explicitly referenced this. Guideline developers require further guidance to enable greater transparency in the reporting of the reasons for discordant recommendations.

**Supplementary Information:**

The online version contains supplementary material available at 10.1186/s12874-023-01895-8.

## Background

Clinical guidelines are part of the landscape of evidence-based healthcare and are considered a key foundation for quality improvement in healthcare in many countries [1, 2]. They are systematically developed statements or recommendations aimed to guide healthcare professionals and patients about appropriate healthcare for specific clinical circumstances [1]. In general, each recommendation is presented with a rating of both its strength and the certainty of the underlying evidence.

The Grading of Recommendations, Assessment, Development and Evaluation (GRADE) approach, used internationally, presents a methodologically rigorous and transparent system for making judgments about the certainty of evidence and strength of recommendations [3]. There are four levels of certainty ratings to rate the certainty of evidence – high, moderate, low, and very low. The GRADE system adopts a considered judgement approach (a structured decision making approach) to making recommendations, and has two categories of recommendations: 1) Strong recommendations confirm confidence that the desirable effects outweigh the undesired consequences and 2) conditional/weak recommendations are made when there is uncertainty regarding potential harms or disadvantages. When the certainty of the evidence is high, Guideline Development Groups (GDGs) are more likely to issue strong recommendations [4, 5].

For the development of trustworthy guidelines there should be concordance between the quality (certainty) of the evidence and the strength of the recommendations. However, guideline developers may need to supplement evidentiary factors (such as quality, quantity, and consistency) with considered judgment (making complex trade-offs between the competing benefits and harms, side effects, and risks of various options for managing the disease or condition) to increase the usage of guideline recommendations in clinical practice [6]. In certain fields, for example, the majority of the evidence may be of low certainty, [7–9] but strong recommendations may be justified when balanced within a considered judgement process. The term discordance has been used to describe differences between the strength of recommendations and the certainty of the evidence [10]. Reflecting this, the GRADE working group has identified five paradigmatic situations (Table 1) in which a strong recommendation could be made based on low or very low certainty of evidence. Two of the five situations (i.e., life-threatening or potential equivalence situations) advocate in favour of the recommendation, while the rest advise against the recommendation [11]. Previous studies indicate varying levels of discordant recommendations internationally, ranging from 12 to 50% [10, 12–14].

**Table 1:** Paradigmatic situation in which a strong recommendation may be made based on low or very low certainty evidence [10, 11]

Paradigmatic situation	Brief explanation	Recommendation	Example situations^a^
Life-threatening (or catastrophical) situation	When low quality evidence suggests benefit in a life-threatening situation (evidence regarding harms can be low or high)	Strong recommendation in favour	Indirect evidence from seasonal influenza suggests that patients with avian influenza may benefit from the use of oseltamivir (low confidence in effect estimates). Given the high mortality of the disease and the absence of effective alternatives, the WHO made a strong recommendation in favour of the use of Oseltamivir rather than no treatment in patients with avian influenza
Potential equivalence, one option clearly less risky or costly	When low quality evidence suggests equivalence of two alternatives, but high-quality evidence of less harm for one of the competing alternatives	Strong recommendation for less harmful/less expensive	H. pylori eradication in patients with early stage Extranodal marginal zone B cell (MALT) lymphoma with H. pylori positive. Low quality evidence suggests that initial H pylori eradication results in similar rates of complete response in comparison to the alternatives of radiation therapy or gastrectomy but with high confidence of less harm/morbidity/cost. Consequently, UpToDate made a strong in favour of H. pylori eradication rather than radiotherapy in patients with MALT lymphoma
Uncertain benefit, certain harm	When very low/ low quality evidence suggests modest benefits and moderate/high quality evidence suggests possibility of harm	Strong recommendation against *(or in favour of the less harmful/less expensive alternative when two are compared)*	In patients with idiopathic pulmonary fibrosis, treatment with azathioprine plus prednisone offers a possible but uncertain benefit in comparison with no treatment. The intervention, however, is associated with a substantial established harm. An international guideline made a recommendation against the combination of corticosteroids plus Azathioprine in patients with idiopathic pulmonary fibrosis
High certainty in similar benefits, one option potentially more risky or costly	When high quality evidence suggests equivalence of two alternatives and low-quality evidence suggests harm in one alternative	Strong recommendation against the intervention with possible greater harm	In women requiring anticoagulation and planning conception or in pregnancy, high confidence estimates suggests similar effects of different anticoagulants. However, indirect evidence (low confidence in effect estimates) suggests potential harm to the unborn infant with oral direct thrombin (eg, dabigatran) and factor Xa inhibitors (eg, rivaroxaban, apixaban). The AT9 guidelines recommended against the use of such anticoagulants in women planning conception or in pregnancy
Potential catastrophic harm	When low to high quality evidence suggests modest benefits and low/ very low-quality evidence suggests possibility of catastrophic harm	Strong recommendation against the intervention *(or in favour of the less harmful/less expensive alternative when two are compared)*	In males with androgen deficiency, testosterone supplementation likely improves quality of life. Low confidence evidence suggests that testosterone increases cancer spread in patients with prostate cancer. The Endocrine Society (USA) made a recommendation against testosterone supplementation in patients with prostate cancer

^**a**^Examples presented were sourced from the MagicApp website

Guideline implementation is a complex process that is often hindered by a variety of individual, organisational, and system level barriers. In particular, communicating the guideline content, both the message of the recommendations themselves and the perception of the evidence being correct and sufficient, deeply affect a guideline’s implementability [15, 16]. Thus, creating strong recommendations based on high certainty evidence and being transparent and clear when making discordant recommendations are crucial in creating trustworthy guidelines [5, 17].

The National Clinical Effectiveness Committee (NCEC) National Clinical Guidelines (NCGs) provide robust evidence-based guidance to inform health care decisions in the Irish health system. The NCEC recommends the use of guideline methodology based on GRADE since 2019 [18]. The aim of this study was to characterize the classification of the strength of recommendations and the certainty of the evidence in Irish NCGs that used the GRADE approach.

## Methods

### Study design and data sources

This study was a cross-sectional analysis of a suite of existing, published NCGs. As of 30^th^ June 2022, there were a total of 29 NCGs published since 2013, by the Department of Health in Ireland, available on their website (https://www.gov.ie/en/collection/c9fa9a-national-clinical-guidelines/).

### Data inclusion

We reviewed all 29 NCGs and included those that described the use of GRADE methodology, either fully or partially, for the grading of recommendations within the guideline.

### Data extraction

For each guideline  we extracted descriptive data including year of publication, the guideline development methodology (e.g., De Novo, ADAPTE [19], Update) and clinical domain. We recorded each individual recommendation using GRADE in full as a unique observation; recommendations that did not use GRADE were excluded. For included recommendations, we extracted the following data:

- Certainty of the evidence (high, moderate, low, and very low)
- Strength of the recommendation (strong and weak/conditional)
- Any justifications provided
  - ○ Did the guidelines reference one of the five paradigmatic situations (Table 1) (yes/no)
- Was a completed Evidence to Decision Making (EtD) framework presented (yes/no).

Specific reference to one of the five paradigmatic situations was noted: 1) Life-threatening (or catastrophical) situation 2) Uncertain benefit, certain harm; 3) Potential equivalence, one option clearly less risky or costly; 4) High certainty in similar benefits, one option potentially more risky or costly; 5) Potential catastrophic harm (Table 1). Where specific reference to any of the five was not presented, the authors classified each of the discordant recommendations as either consistent with one of the five situations (Table 1) or not clear, based on extracted information. Data was extracted from the full guideline document and any relevant appendices (where applicable), including completed EtDs. Ratings of consistency with one of the five paradigmatic situations were linked to the language provided in these documents where possible (see Additional file 1: Appendix 1 for worked example). Authors did not include recommendations based on no included studies within this analysis, given the lack of evidence to base an assessment on.

Data was extracted by one reviewer (MCC) and cross-checked by another (BC). All data was extracted to Microsoft Excel. The reviewers (MCC, BC) resolved all disagreements by discussion.

### Data analysis

Descriptive statistics were used to report the frequency (proportions) of ratings of the certainty of the evidence, strength of the recommendations, and discordant recommendations. The overall rate of discordance across the dataset was calculated as the proportion of all strong recommendations that were based on no studies, low or very low certainty evidence.

## Results

### Summary of included guidelines and recommendations

From the 29 NCEC National Clinical Guidelines available at the time of analysis, eight guidelines published between 2019 and 2022 (Table 2 and Additional file 1: Appendix 2) were identified and all eight used GRADE. As the NCEC has recommended the use of guideline methodology based on GRADE since 2019 [18], the remaining 21 guidelines (published between 2013 and 2019) did not meet the inclusion criteria.

**Table 2:** Descriptive overview of eight included NCGs

Guideline	Number of recommendations	Publication year	Guideline development approach
Irish National Early Warning System (INEWS) V2 [20]	44 • All GRADE	2020	Guideline update
Irish Maternity Early Warning System (IMEWS) V2 [21]	18 • All GRADE	2019	Guideline update
Appropriate prescribing of psychotropic medication for non-cognitive symptoms in people with dementia [22]	21 • All GRADE	2019	ADAPTE and De novo
Nutrition screening and use of oral nutrition support for adults in the acute care setting [23]	17 • 1 GRADE • 16 original rating system	2020	ADAPTE and De novo
Stratification of clinical risk in pregnancy [24]	12 • All GRADE	2020	ADAPTE
Sepsis Management for Adults (including maternity) [25]	93 • 71 GRADE • 18 Best practice statements (ungraded strong recommendations) • 4 no recommendation	2021	ADAPTE
Stop Smoking [26]	18 • All GRADE	2022	ADAPTE and De novo
Unexpected Intraoperative Life Threatening Haemorrhage [27]	17 • All GRADE	2022	ADAPTE

The included eight guidelines covered a broad spectrum of clinical fields, from emergency situations (with four guidelines focused on topics such as early warning scores for clinical deterioration [20, 21], sepsis management [25] and unexpected intraoperative life-threatening haemorrhage [27]), to health behaviours such as smoking cessation [26]. In terms of the guideline development methodologies, six guidelines employed a combination of ADAPTE [19] and de novo development and two were updates of existing guidelines (Table 2).

**Fig. 1:**
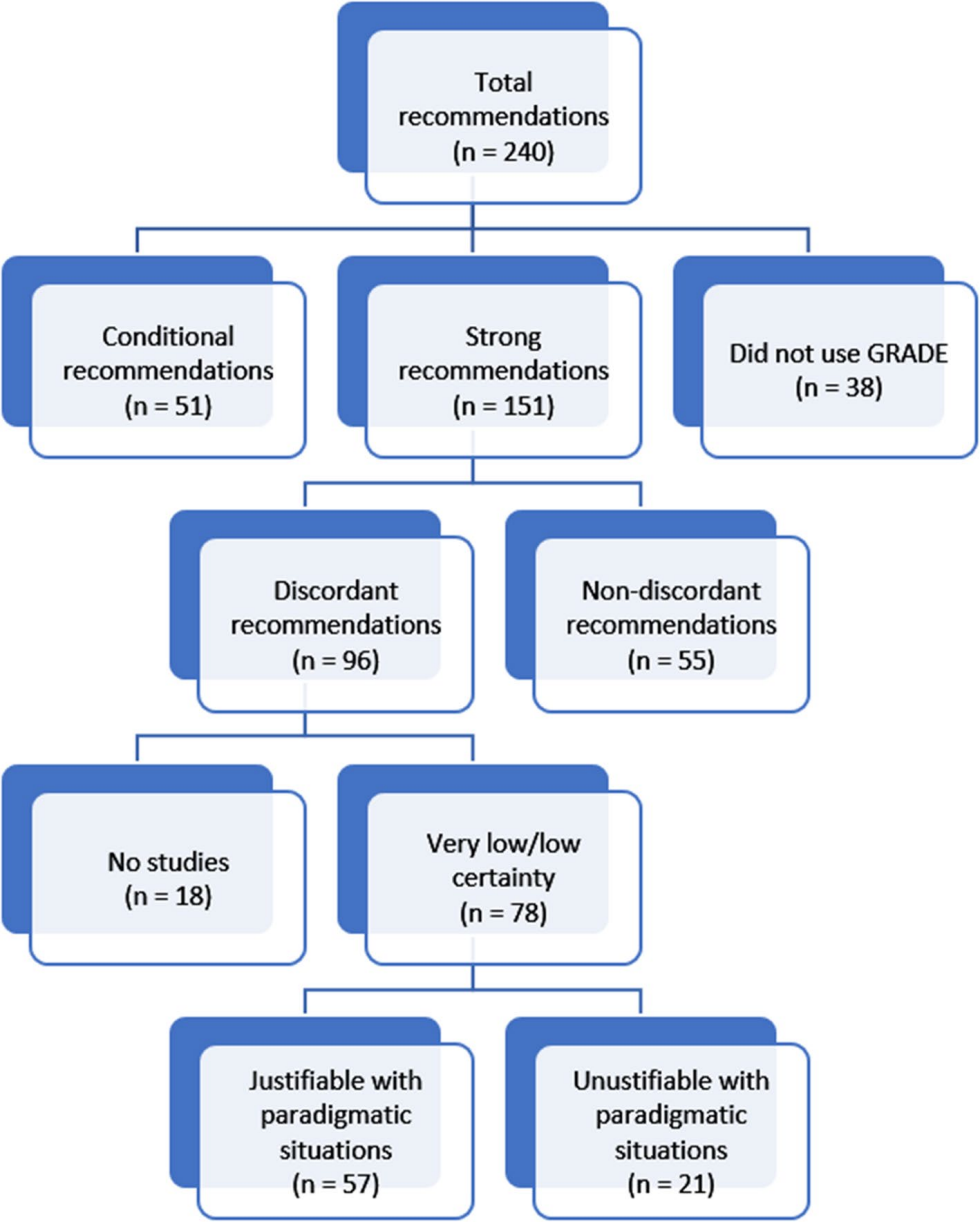
Overview breakdown of all recommendations

There was a total of 240 recommendations across all eight NCGs (median 18, Q1 17, Q3 26.7). A total of 38 recommendations were excluded as they did not use the GRADE approach. This included 18 best practice statements, which are recommended to be designated as such without a formal GRADE rating [28]. Therefore, the final dataset comprised 202 recommendations, of which 151 (74.7%) recommendations were classified as strong and 51 (25.3%) as conditional (Fig. 1).

### Discordant recommendations

Of all strong recommendations (*n* = 151), 55 (36.4%) were based on high or moderate certainty evidence. The remaining 96 (63.6%) were considered discordant recommendations as follows: 60 (39.7%) had very low certainty of evidence, 18 (11.9%) had low certainty, and 18 (11.9%) had no studies included. Of the 51 weak/conditional recommendations, 1 (2.0%) was based on high certainty evidence (Table 3).

**Table 3:** Conditional and strong recommendations stratified by certainty of evidence

Certainty of evidence	Conditional Recommendations *N* = 51 (%)	Strong Recommendations *N* = 151 (%)
No studies	3 (5.9)	18 (11.9)
Very Low	7 (13.7)	60 (39.7)
Low	29 (56.9)	18 (11.9)
Moderate	11 (21.6)	31 (20.5)
High	1 (2.0)	24 (15.9)

**Fig. 2:**
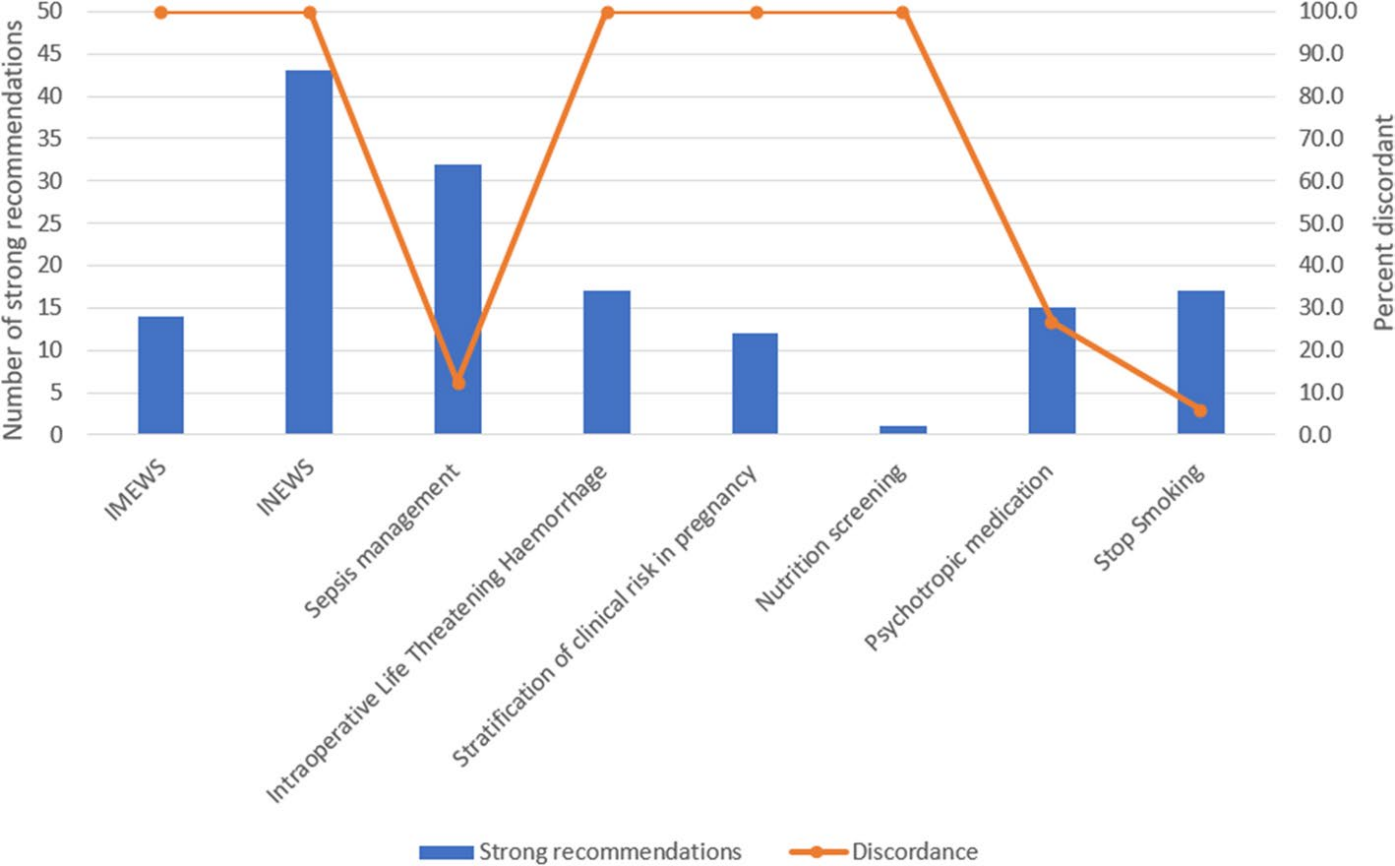
Number of strong recommendations and percent of discordant by individual guidelines. Legend: Blue box—Strong recommendations; Orange line—% discordant

The proportion of discordant recommendations varied across individual guidelines, ranging from 6 to 100% of strong recommendations being based on low or very low certainty evidence or no studies within individual guidelines (Fig. 2, Additional file 1: Appendix 3). Of the five guidelines with 100% of strong recommendations being based on low or very low certainty evidence or no studies, three guidelines had a focus on emergency situations including early warning scores for clinical deterioration [20, 21], and unexpected intraoperative life-threatening haemorrhage [27]) (Fig. 2). The remaining two of these five guidelines related to stratification of clinical risk in pregnancy and nutrition screening in acute care settings, however, this guideline only included one GRADE recommendation.

### Justifications supporting discordant recommendations

Four of the eight included guidelines made a completed EtD framework available. None of the guidelines explicitly referenced any of the five paradigmatic situations to support any identified discordant recommendation. The study team reviewed all strong recommendations that had very low or low certainty (*n* = 78, excluding those based on no studies) to determine if they were consistent with one of the five paradigmatic situations (Additional file 1: Appendix 3). Overall, 55 (73.7%) of these could be mapped to a paradigmatic situation. Of all discordant recommendations justified by a paradigmatic situation (*n* = 55), 63.1% could be retrospectively justified as *‘life-threatening (or catastrophical)’*, 24.6% justified as *‘uncertain benefit, certain harm,’* and 10.5% justified as *‘potential equivalence, one option clearly less risky or costly’* by the study team (Table 4). The remaining strong recommendations that could not be mapped (23/78), largely related to local procedural level recommendations, for example, naming which organisation should take responsibility for certain processes.

**Table 4:** Discordant recommendations justified by paradigmatic situations

Paradigmatic situation	Number of recommendations *N* = 57 (%)
Life-threatening (or catastrophical) situation	36 (63.1)
Uncertain benefit, certain harm	14 (24.6)
Potential equivalence, one option clearly less risky or costly	6 (10.5)
High certainty in similar benefits, one option potentially more risky or costly	1 (1.7)
Potential catastrophic harm	0 (0)

## Discussion

### Summary of results

Among 202 GRADE recommendations from eight national clinical guidelines, the majority of all recommendations were designated as strong, and over half of these strong recommendations were considered discordant. In terms of consistency with one of five paradigmatic situations, we found that the majority of recommendations could be aligned with one of the five. However, while it is reassuring that the majority of strong recommendations based on low or very low certainty evidence were consistent with one of the five paradigmatic situations, this was not explicitly described as such within any of the guideline documents examined.

### Comparison with other studies

Almost three quarters of all recommendations included in this review were designated as strong, which is similar to other studies [12, 14, 29]. Strong recommendations imply that most individuals will be best served by the recommended course of action [30]. Strong recommendations in World Health Organisation (WHO) guidelines have been found to be more frequently adopted than weak or conditional recommendations, [31] underscoring the importance of ensuring that strong recommendations are justified clearly.

Of all strong recommendations included in this review, 64% were considered discordant. Although slightly higher than reported in previous studies, this is reflective of the experiences reported by guideline producing organisations internationally. Among all strong recommendations in Infectious Diseases Society of America guidelines, 47% demonstrated discordance with the quality of evidence [12]. Whereas, a study of WHO recommendations (2007–2012) showed that over 50% of strong recommendations were based on low or very low certainty evidence [13, 14]. An analysis of UpToDate (an electronic medical textbook that uses GRADE) reported that 12% of all strong recommendations were based on low or very low certainty evidence, while an analysis of 215 strong recommendations from critical care medicine guidelines found 32% to be discordant [10].

While we could not explore the reasons for discordant recommendations in this analysis, previous studies have indicated factors that may contribute to such recommendations. For example, there may be scepticism about the value of making conditional recommendations, concerns that conditional recommendations will be ignored, or political considerations such as meeting the needs of ministries of health, and a high certainty or opinion of clinical guideline developers regarding benefits (sometimes warranted, sometimes not) despite rating the evidence as low certainty [32, 33]. While discordant recommendations should be implemented infrequently, these factors may explain the level of discordant recommendations presented in this analysis. Half of the guidelines included pertained to high risk, emergency contexts, and of these three guidelines had 100% discordance. Similar results to ours were reported in a study conducted by the Society of Critical Care Medicine which found that 47 (68.1%) of their 69 strong recommendations were supported by low or very evidence [34]. A robust evidence base is often lacking in areas such as Critical Care and Emergency Medicine [8]. Given the nature of the focus of the Critical Care guidelines, the level of discordance is perhaps not surprising and is reflective of our findings.

In keeping with previous studies, [13, 34] we found that the majority of discordant recommendations could be judged to be consistent with one of the five paradigmatic situations. Again, reflecting that most of the recommendations came from guidelines pertaining to high risk, emergency contexts, the majority of the recommendations could be justified by the *life-threatening (or catastrophical) situation.* Similar to previous reports, we found that none of the guidelines specifically referenced the paradigmatic situations within the accompanying guideline, therefore we were required to make assumptions that may not be reflective of the guideline developers’ original discussions [10, 12]. As a result, we may have under or over estimated the number that may be justifiable.

In this study, 18 strong recommendations were based on no studies. The majority of these were from one guideline, Stratification of Clinical Risk in Pregnancy [24]. This guideline followed an ADAPTE process [19], and in the absence of any suitable guideline being identified to be adapted to the Irish healthcare setting, a modified Delphi approach was undertaken to support guideline development [35]. In certain clinical fields, as was the case with this guideline, applicable evidence may be particularly limited, necessitating the use of expert opinion. However, an analysis of guidelines developed by the American College of Cardiology, the American Heart Association and the American Society of Clinical Oncology (up to 2021) demonstrated that consensus-based guidelines have a greater chance of issuing strong recommendations than evidence-based guidelines [29].

### Strengths and limitations

Strengths of our study include the inclusion of a full suite of National Clinical Guidelines across a wide variety of topics, ranging from emergency situations such as ‘*Unexpected Intraoperative Life Threatening Haemorrhage’*, to health behaviours such as ‘*Stop Smoking*’. In addition, all included NCGs were published within the last three years, reflecting up to date practice. This study was however, limited to a relatively small sample size of eight guidelines—as the NCEC has recommended the use of guideline methodology based on GRADE since 2019 [18], the remaining 21 guidelines (published since 2013) did not meet the inclusion criteria. A large proportion of discordant recommendations originated from a small group of guidelines with several focusing on emergency care, which may influence our overall findings. These topics reflect priorities within the Irish Health system at the time. The guidelines were developed following the NCEC methodology which reflects international best practice, increasing the generalisability of our findings. Although some guidelines were published during the COVID-19 pandemic, they followed standard NCEC methodology and were not part of urgent response guidelines [36]. Two authors (MCC, BC) were involved in data extraction and cross-checking in duplicate, which increases reliability and validity. Furthermore, several authors were also involved in supporting the development of some NCGs, thus providing background knowledge and insights on how these NCGs were developed, allowing for easy navigation of the guideline documents. While involvement in the process could limit the level of objectivity, the primary author conducting data extraction and analysis (MCC) had no prior involvement with any of the included guidelines. Attribution of the recommendations to one of the the paradigmatic situations was done in duplicate but was based on the authors subjective interpretation of the written guideline. The authors did not make an assessment of evidence quality to verify the guideline developers rating of certainty of the evidence.

### Implications

While discordant recommendations should be infrequent in guidelines, they are justifiable options in high-risk situations where stronger evidence is lacking. Our results are reassuring in that the majority of discordant recommendations can be attributed to one of the five paradigmatic situations [10]. However, included guidelines did not explicitly describe this within the guideline nor does the NCEC guideline development manual explicitly require guideline developers to do so [18]. Clinicians using GRADE recommendations need to understand the meaning of the strength of the recommendation, be able to critically appraise the recommendation, and apply trustworthy recommendations according to their strength [32]. Guideline organisations and developers should therefore provide more clarity where strong recommendations based on low or very low evidence certainty are made to increase transparency and overall trustworthiness. This could be achieved through justifications with one of the five paradigmatic situations. The understanding of GRADE by guideline development group members has been identified as a limitation internationally [33] and training of guideline organisations, methodologists and panel members has been identified as important to assure appropriate application of GRADE methodology [37]. The INGUIDE Program is the only currently available course in the world that trains and certifies individuals in guideline development methods [38]. GRADE has been recommended by The Department of Health in Ireland since 2019, however, and these findings may reflect a learning curve with using the approach. Further guidance such as providing evidence-based research training to clinicians who are also guideline developers could help further support them.

### Conclusion

The proportion of discordant recommendations identified in this analysis was higher than some previous international studies (range of all strong recommendations being discordant 30–50%), but similar to other guidelines focused on emergency situations. The majority could be mapped to one of the five paradigmatic situations, but no National Clinical Guideline explicitly referenced this. Guideline developers require further training and guidance in this area to enable them to be more transparent in their reporting of the reasons for discordant recommendations.

## Supplementary Information

**Additional file 1:** **Appendix 1. **Worked example of coding. **Appendix 2. **Summary of all guidelines. **Appendix 3. **Guidelines and the five paradigmatic situations.

## Data Availability

The datasets generated and/or analysed during the current study are available on Open Science Framework (OSF). https://osf.io/ycsd3/?view_only=6972987cac4d4c2e8ab72e1d17de7546 Data are available under the terms of the Creative Commons Attribution 4.0 International license (CC-BY 4.0).

## Abbreviations

EtD: Evidence to Decision Making Framework
GRADE: Grading of Recommendations Assessment, Development and Evaluation
IMEWS: Irish Maternity Early Warning System
INEWS: Irish National Early Warning System
NCEC: National Clinical Effectiveness Committee
NCG: National Clinical Guidelines
WHO: World Health Organisation

## References

[1] Eccles MP, Grimshaw JM, Shekelle P, Schünemann HJ, Woolf S (2012). Developing clinical practice guidelines: target audiences, identifying topics for guidelines, guideline group composition and functioning and conflicts of interest. Implement Sci.

[2] Woolf SH, Grol R, Hutchinson A, Eccles M, Grimshaw J (1999). Clinical guidelines: potential benefits, limitations, and harms of clinical guidelines. BMJ.

[3] Guyatt GH, Oxman AD, Vist GE, Kunz R, Falck-Ytter Y, Alonso-Coello P (2008). GRADE: an emerging consensus on rating quality of evidence and strength of recommendations. BMJ.

[4] Djulbegovic B, Reljic T, Elqayam S, Cuker A, Hozo I, Zhou Q (2019). Structured decision-making drives guidelines panels' recommendations "for" but not "against" health interventions. J Clin Epidemiol.

[5] Djulbegovic B, Hozo I, Li S-A, Razavi M, Cuker A, Guyatt G (2021). Certainty of evidence and intervention's benefits and harms are key determinants of guidelines’ recommendations. J Clin Epidemiol.

[6] Kastner M, Bhattacharyya O, Hayden L, Makarski J, Estey E, Durocher L (2015). Guideline uptake is influenced by six implementability domains for creating and communicating guidelines: a realist review. J Clin Epidemiol.

[7] Zhang S, Wu QJ, Liu SX (2022). A methodologic survey on use of the GRADE approach in evidence syntheses published in high-impact factor urology and nephrology journals. BMC Med Res Methodol.

[8] Conway A, Conway Z, Soalheira K, Sutherland J (2017). High quality of evidence is uncommon in Cochrane systematic reviews in Anaesthesia, critical care and emergency medicine. Eur J Anaesthesiol.

[9] Movsisyan A, Melendez-Torres GJ, Montgomery P (2016). Outcomes in systematic reviews of complex interventions never reached "high" GRADE ratings when compared with those of simple interventions. J Clin Epidemiol.

[10] Agoritsas T, Merglen A, Heen AF, Kristiansen A, Neumann I, Brito JP (2017). UpToDate adherence to GRADE criteria for strong recommendations: an analytical survey. BMJ Open.

[11] Andrews JC, Schünemann HJ, Oxman AD, Pottie K, Meerpohl JJ, Coello PA (2013). GRADE guidelines: 15. Going from evidence to recommendation-determinants of a recommendation's direction and strength. J Clin Epidemiol.

[12] Miles KE, Rodriguez R, Gross AE, Kalil AC (2021). Strength of recommendation and quality of evidence for recommendations in Current Infectious Diseases Society of America Guidelines. Open Forum Infect Dis.

[13] Alexander PE, Brito JP, Neumann I, Gionfriddo MR, Bero L, Djulbegovic B (2016). World Health Organization strong recommendations based on low-quality evidence (study quality) are frequent and often inconsistent with GRADE guidance. J Clin Epidemiol.

[14] Alexander PE, Bero L, Montori VM, Brito JP, Stoltzfus R, Djulbegovic B (2014). World Health Organization recommendations are often strong based on low confidence in effect estimates. J Clin Epidemiol.

[15] Francke AL, Smit MC, de Veer AJ, Mistiaen P (2008). Factors influencing the implementation of clinical guidelines for health care professionals: a systematic meta-review. BMC Med Inform Decis Mak.

[16] Correa VC, Lugo-Agudelo LH, Aguirre-Acevedo DC, Contreras JAP, Borrero AMP, Patiño-Lugo DF (2020). Individual, health system, and contextual barriers and facilitators for the implementation of clinical practice guidelines: a systematic metareview. Health Res Policy Syst.

[17] Pronovost PJ (2013). Enhancing Physicians’ Use of Clinical Guidelines. JAMA.

[18] Department of Health (2019). How to develop a National Clinical Guideline: A manual for guideline developers.

[19] The ADAPTE Collaboration (2009). DAPTE Resource Toolkit for guideline adaptation Version 2.0.

[20] Department of Health (2020). Irish National Early Warning System (INEWS) Version 2 (NCEC National Clinical Guideline No 1).

[21] Department of Health (2019). Irish Maternity Early Warning System (IMEWS) Version 2 (NCEC National Clinical Guideline No 4).

[22] Department of Health (2019). Appropriate prescribing of psychotropic medication for non-cognitive symptoms in people with dementia (NCEC National Clinical Guideline No 21).

[23] Department of Health (2020). Nutrition screening and use of oral nutrition support for adults in the acute care setting (NCEC National Clinical Guideline No 22).

[24] Department of Health (2022). Stratification of clinical risk in pregnancy (NCEC National Clinical Guideline No 23).

[25] Department of Health (2021). Sepsis Management for Adults (including maternity) (NCEC National Clinical Guideline No 26).

[26] Department of Health (2022). Stop Smoking (NCEC National Clinical Guideline No 28).

[27] Department of Health (2022). Unexpected Intraoperative Life Threatening Haemorrhage (NCEC National Clinical Guideline No 29).

[28] Guyatt GH, Schünemann HJ, Djulbegovic B, Akl EA (2015). Guideline panels should not GRADE good practice statements. J Clin Epidemiol.

[29] Yao L, Ahmed MM, Guyatt GH, Yan P, Hui X, Wang Q (2021). Discordant and inappropriate discordant recommendations in consensus and evidence based guidelines: empirical analysis. BMJ.

[30] Schünemann H, Brozek J, Guyatt G, Oxman AD, editors. Handbook for grading the quality of evidence and the strength of recommendations using the GRADE approach. Updated October 2013. https://gdt.gradepro.org/app/handbook/handbook.html#h.w29yp7vuyzwo: GRADE working Group; 2013.

[31] Nasser SM, Cooke G, Kranzer K, Norris SL, Olliaro P, Ford N (2015). Strength of recommendations in WHO guidelines using GRADE was associated with uptake in national policy. J Clin Epidemiol.

[32] Alexander PE, Gionfriddo MR, Li S-A, Bero L, Stoltzfus RJ, Neumann I (2016). A number of factors explain why WHO guideline developers make strong recommendations inconsistent with GRADE guidance. J Clin Epidemiol.

[33] Alexander PE, Li SA, Gionfriddo MR, Stoltzfus RJ, Neumann I, Brito JP (2016). Senior GRADE methodologists encounter challenges as part of WHO guideline development panels: an inductive content analysis. J Clin Epidemiol.

[34] Sims CR, Warner MA, Stelfox HT, Hyder JA (2019). Above the GRADE: Evaluation of Guidelines in Critical Care Medicine. Crit Care Med..

[35] Clyne B, Tyner B, O'Neill M, Jordan K, Carty PG, Phillips MK (2022). ADAPTE with modified Delphi supported developing a national clinical guideline: stratification of clinical risk in pregnancy. J Clin Epidemiol.

[36] Akl EA, Morgan RL, Rooney AA, Beverly B, Katikireddi SV, Agarwal A (2021). Developing trustworthy recommendations as part of an urgent response (1–2 weeks): a GRADE concept paper. J Clin Epidemiol.

[37] Dixon C, Dixon PE, Sultan S, Mustafa R, Morgan RL, Murad MH (2020). Guideline developers in the United States were inconsistent in applying criteria for appropriate grading of recommendations, assessment, development and evaluation use. J Clin Epidemiol.

[38] Piggott T, Baldeh T, Akl EA, Junek M, Wiercioch W, Schneider R (2021). Supporting effective participation in health guideline development groups: the guideline participant tool. J Clin Epidemiol.

