# Supplementary material for: Strong recommendations from low certainty evidence: a cross-sectional analysis of a suite of national guidelines
Source: BMC Med Res Methodol. 2023 Mar 25;23:68. doi: 10.1186/s12874-023-01895-8 (PMC10039768; doi:10.1186/s12874-023-01895-8)
Supplement: Supplementary file 1 — Additional file 1: Appendix 1. Worked example of coding. Appendix 2. Summary of all guidelines. Appendix 3. Guidelines and the five paradigmatic situations. [file 12874_2023_1895_MOESM1_ESM.docx]

**Additional file 1: Appendix 1** Worked example of coding

| **Recommendation** | **Quality of evidence** | **Strength of recommendation** | **Justification** | **Paradigmatic situations selected** | **Rationale** |
| --- | --- | --- | --- | --- | --- |
| All hospitals must have the National Life Threatening Haemorrhage Management Poster prominently on display in the operating theatre. All hospitals must also have an underpinning life threatening haemorrhage policy & procedure/protocol which incorporates the recommendations of this guideline. All clinical, laboratory and support staff to maintain their competency must be familiar with the contents of the life threatening haemorrhage protocol/ procedure. | Low | Strong | A survey undertaken by the GDG of all blood transfusion laboratories across the country identified gaps in the availability of a Life Threatening Haemorrhage Protocol/Procedure. The survey also identified that in addition to the Protocol, most hospitals summarised information from the Protocol in a single page format (one page poster). Many hospitals had a poster available in theatre but a lack of standardisation in the content displayed on this poster was apparent. Having a standard format will be helpful for theatre staff and all rotating trainees. | Life-threatening (or catastrophical) situation | - Life threatening outcome: Life Threatening Haemorrhage |

**Additional file 1: Appendix 2.** Summary of all guidelines

| **NCEC Guidelines with GRADE** | **Total number of recommendations using GRADE** | **Strength of**  **Recommendations (%)** | | **Discordant recommendations (%)** | **Completed EtD published with guideline** |
| --- | --- | --- | --- | --- | --- |
|  |  | ***Conditional*** | ***Strong*** |  |  |
| **INEWS** | | | | **43/43 (100.0)** | **No** |
| *No studies* | 0 | 0 | 0 |  |  |
| Very low | 44 | 1 | 43 |  |  |
| Low | 0 | 0 | 0 |  |  |
| Moderate | 0 | 0 | 0 |  |  |
| High | 0 | 0 | 0 |  |  |
| **Total** | **44** | **1 (2.3)** | **43 (97.7)** |  |  |
| **IMEWS** | | | | **14/14 (100.0)** | **Yes** |
| *No studies* | 9 | 3 | 6 |  |  |
| Very low | 9 | 1 | 8 |  |  |
| Low | 0 | 0 | 0 |  |  |
| Moderate | 0 | 0 | 0 |  |  |
| High | 0 | 0 | 0 |  |  |
| **Total** | **18** | **4 (22.2)** | **14 (77.3)** |  |  |
| **Prescribing of psychotropic medication** | | | | **4/15 (26.6)** | **No** |
| *No studies* | 0 | 0 | 0 |  |  |
| Very low | 0 | 0 | 0 |  |  |
| Low | 5 | 1 | 4 |  |  |
| Moderate | 11 | 4 | 7 |  |  |
| High | 5 | 1 | 4 |  |  |
| **Total** | **21** | **6 (28.6)** | **15 (71.4)** |  |  |
| **Nutrition screening** | | | | **1/1 (100.0)** | **Yes** |
| *No studies* | 0 | 0 | 0 |  |  |
| Very low | 0 | 0 | 0 |  |  |
| Low | 1 | 0 | 1 |  |  |
| Moderate | 0 | 0 | 0 |  |  |
| High | 0 | 0 | 0 |  |  |
| **Total** | 1 | **0 (0.0)** | **1 (100.0)** |  |  |
| **Stratification of clinical risk** | | | | **12/12 (100.0)** | **No** |
| *No studies* | 12 | 0 | 12 |  |  |
| *Very low* | 0 | 0 | 0 |  |  |
| *Low* | 0 | 0 | 0 |  |  |
| *Moderate* | 0 | 0 | 0 |  |  |
| *High* | 0 | 0 | 0 |  |  |
| **Total** | **12** | **0 (0.0)** | **12 (100.0)** |  |  |
| **Sepsis Management for Adults (including maternity)** | | | | **4/32 (12.5)** | **No** |
| *No studies* | 0 | 0 | 0 |  |  |
| Very low | 5 | 5 | 0 |  |  |
| Low | 31 | 27 | 4 |  |  |
| Moderate | 28 | 7 | 21 |  |  |
| High | 7 | 0 | 7 |  |  |
| **Total** | **71** | **39 (54.9)** | **32 (45.1)** |  |  |
| **Stop Smoking** |  |  |  | **1/17 (5.9)** | **Yes** |
| *No studies* | 0 | 0 | 0 |  |  |
| Very low | 0 | 0 | 0 |  |  |
| Low | 2 | 1 | 1 |  |  |
| Moderate | 3 | 0 | 3 |  |  |
| High | 13 | 0 | 13 |  |  |
| **Total** | **18** | **1 (5.6)** | **17 (94.4)** |  |  |
| **Unexpected Intraoperative Life Threatening Haemorrhage** | | | | **17/17 (100.0)** | **Yes** |
| *No studies* | 0 | 0 | 0 |  |  |
| Very low | 9 | 0 | 9 |  |  |
| Low | 8 | 0 | 8 |  |  |
| Moderate | 0 | 0 | 0 |  |  |
| High | 0 | 0 | 0 |  |  |
| **Total** | **17** | **0 (0.0)** | **17 (100.0)** |  |  |

Abbreviations:

**Additional file 1: Appendix 3.** Guidelines and the five paradigmatic situations

| **Guidelines** | **Five paradigmatic situations*** | | | | |  |
| --- | --- | --- | --- | --- | --- | --- |
|  | **Life-threatening**  **(or catastrophical) situation**  **N =36** | **Potential equivalence, one option clearly less risky or costly**  **N = 6** | **Uncertain benefit, certain harm**  **N = 14** | **High certainty in similar benefits, one option potentially more risky or costly**  **N =1** | **Potential catastrophic harm**  **N = 0** | **Not reported/**  **unclear**  **N = 21** |
| Irish National Early Warning System (INEWS) V2 (21) | 14 | 4 | 6 | 0 | 0 | 20 |
| Irish Maternity Early Warning System (IMEWS) V2 (20) | 3 | 1 | 2 | 0 | 0 | 1 |
| Appropriate prescribing of psychotropic medication for non-cognitive symptoms in people with dementia (36) | 0 | 0 | 3 | 1 | 0 | 0 |
| Nutrition screening and use of oral nutrition support for adults in the acute care setting (37) | 0 | 0 | 1 | 0 | 0 | 0 |
| Stratification of clinical risk in pregnancy (32) | 0 | 0 | 0 | 0 | 0 | 0 |
| Sepsis Management for Adults (including maternity) (22) | 2 | 1 | 1 | 0 | 0 | 0 |
| Stop Smoking (24) | 0 | 1 | 1 | 0 | 0 | 0 |
| Unexpected Intraoperative Life Threatening Haemorrhage (23) | 17 | 0 | 0 | 0 | 0 | 0 |
